# ﻿From morphology to molecules: A comprehensive study of a novel *Derris* species (Fabaceae) with a rare flowering habit and reddish leaflet midribs, discovered in Peninsular Thailand

**DOI:** 10.3897/phytokeys.237.112860

**Published:** 2024-01-15

**Authors:** Punvarit Boonprajan, Charan Leeratiwong, Yotsawate Sirichamorn

**Affiliations:** 1 Department of Biology, Faculty of Science, Silpakorn University, Sanam Chandra Palace Campus, Nakhon Pathom 73000, Thailand Silpakorn University Nakhon Pathom Thailand; 2 Division of Biological Science, Faculty of Science, Prince of Songkla University, Songkhla 90112, Thailand Prince of Songkla University Songkhla Thailand

**Keywords:** Anatomy, *Derris*, HPLC fingerprint, molecular phylogeny, morphology, phytochemical

## Abstract

*Derrisrubricosta* Boonprajan & Sirich., **sp. nov.**, a new species of the genus *Derris* Lour. (Fabaceae) was discovered in Peninsular Thailand. The overall morphology demonstrates that the species most resembles *D.pubipetala*. Nevertheless, the species has several autapomorphies differentiating it from other *Derris* species, e.g., the presence of reddish midribs of the mature leaflets, sparsely hairy stamen filaments, prominent hairs at the base of the anthers, and presence of glandular trichomes along the leaflet midrib. Additionally, HPLC fingerprints of this species showed a distinction from *D.pubipetala* by the absence of phytochemical compound peaks after 13 min. Retention Time (RT). Results from molecular phylogenetic analyses also strongly supported the taxonomic status as a new species.

## ﻿Introduction

The genus *Derris* Lour. is a papilionoid legume, comprising approximately 50 species (Adema 2000, 2003; Sirichamorn 2020; POWO 2023). It is a taxonomically complex genus in the tribe Millettieae of Fabaceae. Species of *Derris* are important sources of a toxic substance, Rotenone, which is traditionally used as a fish poison and commercially used as an insecticide. In addition, many species of *Derris* are used as local medicines for a variety of ailments in many South-East Asian countries (Hamid 1999).

*Derris* was first described by Loureiro (1790), based on the type species *D.trifoliata* Lour. Bentham (1860) recognised the genus in a taxonomically broad sense and his concept has been widely followed since then. After revisions of the genus based mainly on morphological and molecular phylogenetic analyses, the circumscription of *Derris* became taxonomically narrower (Geesink 1985; Adema 2000, 2003; Sirichamorn et al. 2012a; Sirichamorn et al. 2012b; Sirichamorn et al. 2014b).

All *Derris* species are lianas, with imparipinnate leaves and opposite leaflets, its inflorescences are pseudoracemes/pseudopanicles, or rarely panicles, the androecium is monadelphous and the stamens generally glabrous, the fruits are indehiscent, laterally compressed, leathery pods, usually with marginal wings. However, tardily dehiscent, wingless, and inflated pods were also reported in *D.montana* Benth. (Sirichamorn et al. 2012a) and recently in *D.taiwaniana* (Hayata) Z.Q.Song and *D.entadoides* (Z.Wei) Z.Q.Song (Song and Pan 2022). Some genera in the tribe Millettieae such as the Asiatic *Aganope* Miq. and *Brachypterum* (Wight & Arn.) Benth. or the American *Deguelia* Aubl. are, however, morphologically very similar to *Derris* and are sometimes referred to as *Derris*-like plants (Sirichamorn et al. 2012a). Species of *Derris* are found mainly in South-East Asia. Seventeen species were reported in the Flora of Thailand (Sirichamorn 2020). In addition, previously unknown Thai taxa have been discovered in different parts of the country.

Thailand is a part of the ancestral center of *Derris* (Sirichamorn et al. 2014a, sits at the nexus of three key floristic regions: Indo-Chinese, Indo-Burmese and Malesian elements. Southern Thailand, positioned on the Malay Peninsula and bordered northward by the Kra Isthmus, predominantly lies within the Tenasserim-South Thailand semi-evergreen rainforest ecoregion. This stretch spans the western Andaman Sea coast and the eastern Gulf of Thailand coast. Conversely, the southernmost border with Malaysia falls under the Peninsular Malaysian rainforest and montane rainforest ecoregions (Wikramanayake et al. 2002). Areas such as the Hala-Bala Wildlife Sanctuary, straddling the Thai-Malaysian border, house an array of rare and indigenous flora and fauna (ICEM 2003). A variety of new plant taxa have been discovered and reported from the area in the past two decades including the legume species such as *Derrisglabra* Sirich. (Sirichamorn et al. 2012a) and *Millettiacalcicola* Mattapha, G.P. Lewis & Hawkins (Mattapha et al. 2023).

During a field expedition in 2019, the authors of this article discovered and collected an unrecognised “*Derris*-like” species along the stream at Pha Dam Forest Ranger Unit (Ton Nga Chang Wildlife Sanctuary), Songkhla province. However, the plant showed some unusual morphological characteristics compared with other known species of *Derris* and *Derris*-like taxa. During comprehensive field surveys in the following years, two more populations were found by the stream at the entrance of Tone Prew waterfall (Ton Nga Chang Wildlife Sanctuary, also in Songkhla province) and Krung Ching waterfall (in Khao Luang National Park in Nakhon Si Thammarat province). The latter two populations had very similar vegetative characters, but unfortunately were never observed producing flowers. The flowering specimen from Pha Dam Forest Ranger Unit only once produced inflorescences in 2019, but no seed pod was produced. The specimens were compared with the type specimens of all *Derris*-like taxa, including the descriptions in several taxonomic publications, including the Flora of Thailand (Sirichamorn 2020), the Flora of the Malay Peninsula (Ridley 1922), the Flora of China (Chen and Pedley 2010), the Flora of India (Thothathri 1982), the Flora of British India (Baker 1878) and a taxonomic revision of the genus in India (Thothathri 1961). Preliminary morphological studies pointed to the plant being an undescribed species of *Derris*.

The main objectives of this work were to investigate and verify the exact taxonomic status of this unknown *Derris*-like species using more critical macro- and micro-morphological studies, molecular phylogenetic analyses, and phytochemical evidence from high-performance liquid chromatography (HPLC) fingerprinting. A full species description and revised key to the species of *Derris* in Thailand are provided, together with photographs and a line drawing of the new taxon.

## ﻿Materials and methods

### ﻿Taxon collection, preparation, and taxonomic study

Samples from three localities of the putative new taxon, and three samples of *D.pubipetala* from three different localities (represented by accessions nos. 1 to 6 in Table 1), were collected for morphological, anatomical, phytochemical, and molecular investigation. Voucher specimens were deposited in the BKF herbarium and duplicates were distributed to other herbaria. Three mature leaflets per accession were fixed in 70% ethyl alcohol for anatomical study. Young leaves of the three putative new taxon samples were collected and stored in silica gel for later DNA extraction. Stems and roots were also collected, cleaned, cut into smaller pieces and then dried at 50–60 °C in a hot air oven, and then stored at room temperature for phytochemical study.

**Table 1.** Species, localities, and vouchers of the material in Thailand used for morphological and phytochemical analysis.

Accession No.	Species	Locality	Voucher specimen	Herbarium
1	*Derris* sp.	Pha Dam Forest Ranger Unit, Ton Nga Chang Wildlife Sanctuary, Padang Besar sub-district, Sadao district, Songkhla Province (Locality code; SS)	C. Leeratiwong 19-1666	BKF
2	*Derris* sp.	Tone Prew waterfall, Ton Nga Chang Wildlife Sanctuary, Kamphaeng Phet sub-district, Rattaphum district, Songkhla Province (Locality code; RS)	YSM2021-15	BKF
3	*Derris* sp.	Krung Ching waterfall, Nopphitam sub-district, Nopphitam district, Nakhon Si Thammarat Province (Locality code; NN)	YSM2021-16	BKF
4	*D.pubipetala*	Surat Thani, Ko Samui district, Ang Thong sub-district (Hin Lad Waterfall)*	Leeratiwong et al. 18-1192	PSU
5	*D.pubipetala*	Nakhon Si Thammarat, Khanom district, Khanom sub-district (Samedchun Waterfall)	YSM2022-5	BKF
6	*D.pubipetala*	Nakhon Si Thammarat, Khanom district, Khanom sub-district (Hin Lad Waterfall)*	YSM2022-6	BKF

Note: ^*^The name of both waterfalls is the same, but they are different locations.

Voucher specimens were examined using a stereomicroscope. The species description was prepared following the format of the Flora of Thailand (Sirichamorn 2020). Morphological measurements and comparison of specimens with (type) specimens of *Derris*-like plants housed in Thai herbaria (BK, BKF, and PSU) or available as online digital images (K, L, and P) were carried out.

### ﻿Scanning electron microscopy (SEM)

Three lateral leaflets of mature leaves for each accession were used. Samples of 5 × 5 mm were taken from the center of the leaflets (from midrib to margin, including the midrib). Sections were cleaned, dehydrated in a series of ethyl alcohol, dried by critical point drying (CPD) in liquid CO_2_, and preserved in a desiccator for subsequent observation by SEM. Samples were mounted directly on aluminum stubs using double-sided carbon tape, then sputter-coated with gold using an SPI module sputter coater. The samples were photographed using a Tescan Mira3 scanning electron microscope (SEM) at the Scientific and Technological Equipment Center, Faculty of Science, Silpakorn University.

### ﻿Anatomical study of leaves

Leaf epidermis was studied using the leaf scraping technique (modified from Johansen 1940). Mesophyll was scraped from the upper and lower surfaces with a razor blade, followed by epidermal bleaching using 10% sodium hypochlorite. Samples were cleaned three times in distilled water, then stained with 1% Safranin-O before washing again and dehydrating in a graded series of ethyl alcohol and a series of xylene. Samples were mounted on slides using DePeX mounting media (VWR international Ltd., England).

Three leaflets from 3 mature leaves for each accession were sectioned by hand to produce transverse sections, then stained with 1% Safranin-O, dehydrated in a graded series of ethyl alcohol and series of xylene. Finally, the sections were mounted on slides using DePeX. All leaf epidermal surfaces and sectioned parts were digitally photographed with an Olympus BX53 microscope with a DP27 camera attachment. Each leaf anatomical character was measured using ImageJ (Rueden et al. 2017).

### ﻿Phytochemical analyses

Dried stems and roots of each accession were ground separately into a powder using a high-speed blender. The maceration process (modified from da Costa et al. 2016) involved soaking 5 grams of each plant sample in 250 mL of Dichloromethane in the dark at room temperature for 72 hrs. After maceration, the solvent extract of each accession was then ﬁltered and subsequently evaporated in a dark fume hood at room temperature for 24 hrs. The crude extract was transferred into a sealed plastic tube, protected from light and stored in a refrigerator at 0–5 °C for further analysis.

The qualitative phytochemical analysis of rotenone and deguelin was analyzed using high-performance liquid chromatography (Agilent Technologies, Germany) consisting of a 1260 Infinity II LC system controller ﬁtted with 1260 Infinity II Quaternary, 1260 Infinity Binary pump, 1260 Infinity II degasser, and 1260 Infinity II Diode Array UV-Visible detector. An Agilent 5 TC-C18 (4.6 × 150 mm, particle size 5 µm) reversed-phase analytical column was used. The isocratic method was performed for separating chemical substances using a mobile phase of acetonitrile/water (60:40; v/v) for 35 min. The injection volume was 10 µL, and the flow rate was 1.0 mL/min. The liquid chromatography system had to be stabilised for 15 min with the mobile phase before injecting the analyte. The analysis was performed at a wavelength of 294 nm. The control and data elaboration used Agilent OpenLAB ChemStation Edition.

Approximately 0.01 g of crude extract was dissolved and diluted with 1 mL of acetonitrile in a 2 mL microcentrifuge tube. The extracted solution was filtered using a syringe filter nylon membrane of 0.22 µm pore size. The chemical patterns of the two mains chemical markers were carried out using the HPLC system.

### ﻿Molecular analyses

Taxon sampling for this study were selected based on the phylogeny reconstructed by Sirichamorn et al. (2012a) (Suppl. material 1). Additional DNA samples were extracted from dried young leaves of the three populations of the putative new species using the DNeasy Plant mini kit and modiﬁed protocol (Qiagen, Hilden, Germany). Two chloroplast regions [*trnL*-*F* intergenic spacer (IGS) and *trnK*-*matK*] and one nuclear region [the ribosomal internal transcribed spacer (ITS/5.8S)] were amplified using universal primers (Table 2). PCR reagents were carried out in a 25 µL reaction mixture which contained 1 µL (10 µM) of each forward and reverse primer, 12.5 µL GoTag Green Master Mix (Promega), 2 µL (< 250 ng) of total DNA and Nuclease-free water to 25 µL. PCR conditions for *trnL*-*F*IGS and *trnK*-*matK* followed a modified protocol of Hu et al. (2000). The ITS/5.8S region was amplified following Wojciechowski et al. (1993, 1999). Quality, quantity, and size of PCR products were tested by gel electrophoresis and genomic absorption. Samples were sent to Celemics, Inc. (http://www.celemics.com) for purifying and sequencing. Each purified fragment was treated using a Barcode-Tagged Sequencing (BTSeq) technique of Next-Generation Sequencing (NGS) (CELEMICS, Seoul, Republic of Korea) technology for automated dsDNA sequencing. All newly generated sequences in this study have been deposited in GenBank (Suppl. material 1).

**Table 2.** Sequences of the primers used for PCR amplification and sequencing.

Primers	Amplified region	Direction	Sequence (5 ′ to 3 ′)	References
trnKIL	*trnK*-*matK* (Ch)	forward	CTC AAT GGT AGA GTA CTC G	Hu et al. 2000
trnK2R	*trnK*-*matK* (Ch)	reverse	AAC TAG TCG GAT GGA GTA G	Hu et al. 2000
e	*trnL*-*F*IGS (Ch)	forward	GGT TCA AGT CCC TCT ATC CC	Taberlet et al. 1991
f	*trnL*-*F*IGS (Ch)	reverse	ATT TGA ACT GGT GAC ACG AG	Taberlet et al. 1991
ITS1	ITS/5.8S (Nr)	forward	TCC GTA GGT GAA CCT GCG G	White et al. 1990
ITS4	ITS/5.8S (Nr)	reverse	TCC TCC GCT TAT TGA TAT GC	Wojciechowski et al. 1993

Ch = chloroplast marker; Nr = nuclear marker.

Sequence alignments were performed using the program Bioedit v. 7.0.9 (Hall 1999) with the CLUSTAL W multiple alignment (default settings; Thompson et al. 1994) together with subsequent manual adjustment. Sequences of each marker were firstly aligned separately. Then, the combined data matrix was made by concatenating those already aligned datasets. Phylogenetic relationship was reconstructed using Maximum Parsimony (MP), Maximum Likelihood (ML), and Bayesian Inference (BI). For MP analyses, phylogenetic trees were constructed with the program PAUP* v. 4.0a169 (Swofford 2002) under heuristic search with 10,000 replicates of random taxon additions. The process employed Tree Bisection-Reconnection (TBR) branch swapping and Multrees activated, with all parsimonious trees saved. Bootstrap percentage analysis was used for the evaluation of MP clade support and calculated using the same settings (Felsenstein 1985). Maximum Parsimony Bootstrap Support (MPBS) is described as high (85–100%), moderate (75–84%), low (50–74%), or none (< 50%). The jModelTest v. 2 (Darriba et al. 2012) on the CIPRES web portal was used to find the best-fit substitution model chosen by the Akaike Information Criterion (AIC) scores (Akaike 1974). The General Time Reversible (GTR) (Tavaré 1985) nucleotide substitution model with a gamma distribution for among-site rate variation was selected for all DNA regions. ML analyses were performed for the combined data sets using the program IQ-TREE v. 2.2.0 (Nguyen et al. 2015) under GTR+G partition models implemented with the “-p” command. Bootstrap (Felsenstein 1985) clade support was calculated using a non-parametric bootstrap resampling with 2,000 replicates. Maximum Likelihood Bootstrap Support (MLBS) values are described as high (85–100%), moderate (75–84%), low (50–74%), or none (< 50%). Bayesian Markov Chain Monte Carlo (MCMC) (Yang and Rannala 1997) phylogenetic analyses were reconstructed using the program MrBayes v. 3.2.7a (Ronquist et al. 2012) via the CIPRES Science Gateway v.3.3 (Miller et al. 2010). The majority-rule consensus tree was started from random trees and run for 10,000,000 generations until stationarity, with MCMC sampled every 1,000 generations. The first 25% of all trees were discarded as burn-in and each BI clade was supported by posterior probabilities (PP) estimation from the remaining sampled trees. PPs are described as high (0.95–1), moderate (0.9–0.94), low (0.5–0.89), or none (< 0.5) support.

### ﻿Morphological study

**Figure 1.**
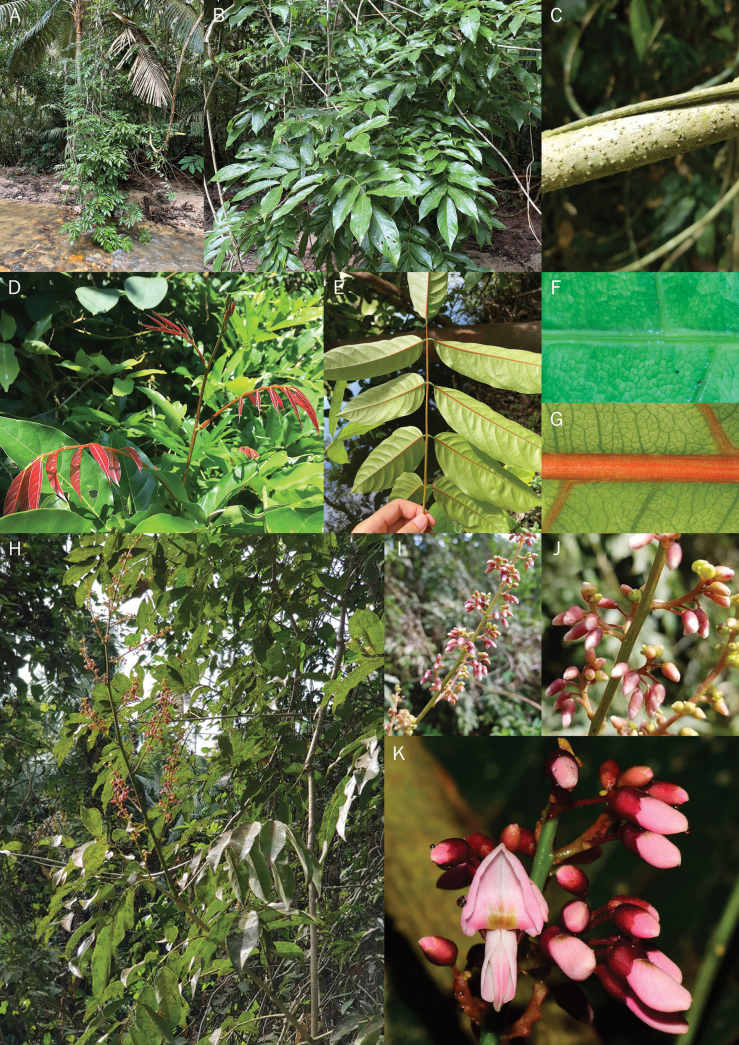
*Derrisrubricosta*, sp. nov. **A** habit and habitat **B** leaves **C** stem **D** young reddish leaflets **E** reddish midribs of mature leaflets **F**, **G** close-up of midrib on adaxial and abaxial surface respectively **H** inflorescences and foliage **I**, **J** close-up of inflorescence, showing flower buds and brachyblasts **K** close-up of flower and buds. All photos were taken at Pha Dam Forest Ranger Unit in Songkhla province. Photos by Punvarit Boonprajan (**A**, **B**, **D**–**G**) and Charan Leeratiwong (**C**, **H**–**K**).

**Figure 2.**
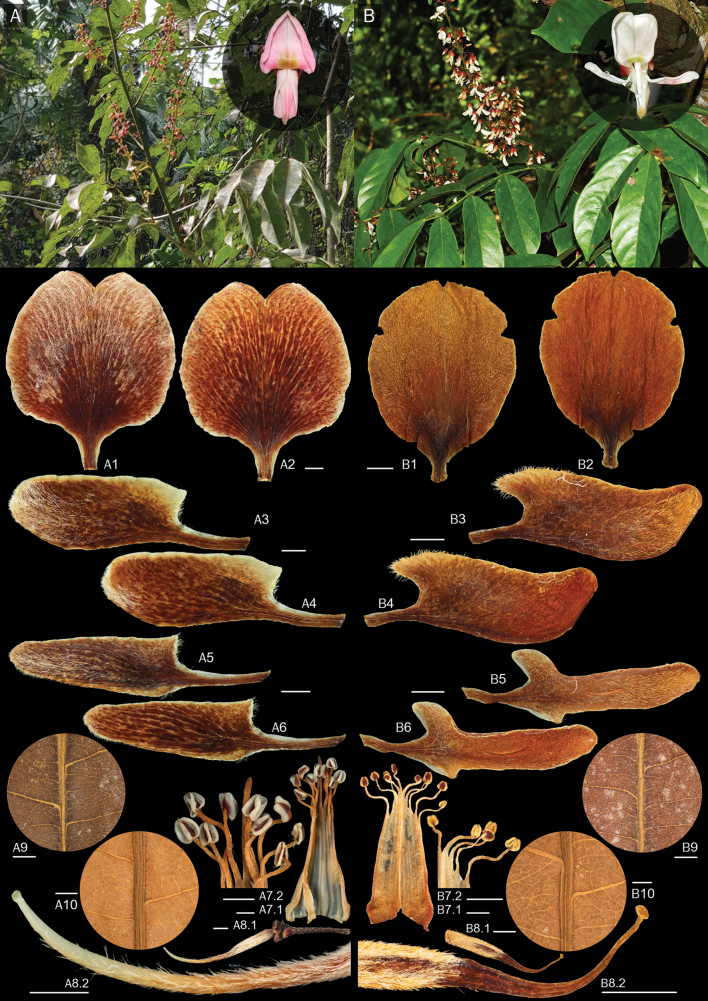
Comparative macro- and micro-morphological characters of leaflets and flowers of *Derrisrubricosta* (**A, A1–A10**) and *D.pubipetala* (**B**, **B1–B10**) **A, B** a branch with leaves and inflorescences **A1, B1** outer surface and **A2, B2** inner surface of standard petal **A3, B3** outer surface and **A4, B4** inner surface of keel petals **A5, B5** outer surface and **A6, B6** inner surface of wing petals **A7.1, B7.1** stamens and **A7.2, B7.2** close-up stamens **A8.1, B8.1** pistil and **A8.2, B8.2** close-up pistil apex **A9, B9** adaxial and **A10, B10** abaxial leaflet surfaces. Photos by Charan Leeratiwong (**A, B**) and Punvarit Boonprajan (**A1–A10, B1–B10**). Scale bars: 1 mm.

**Figure 3.**
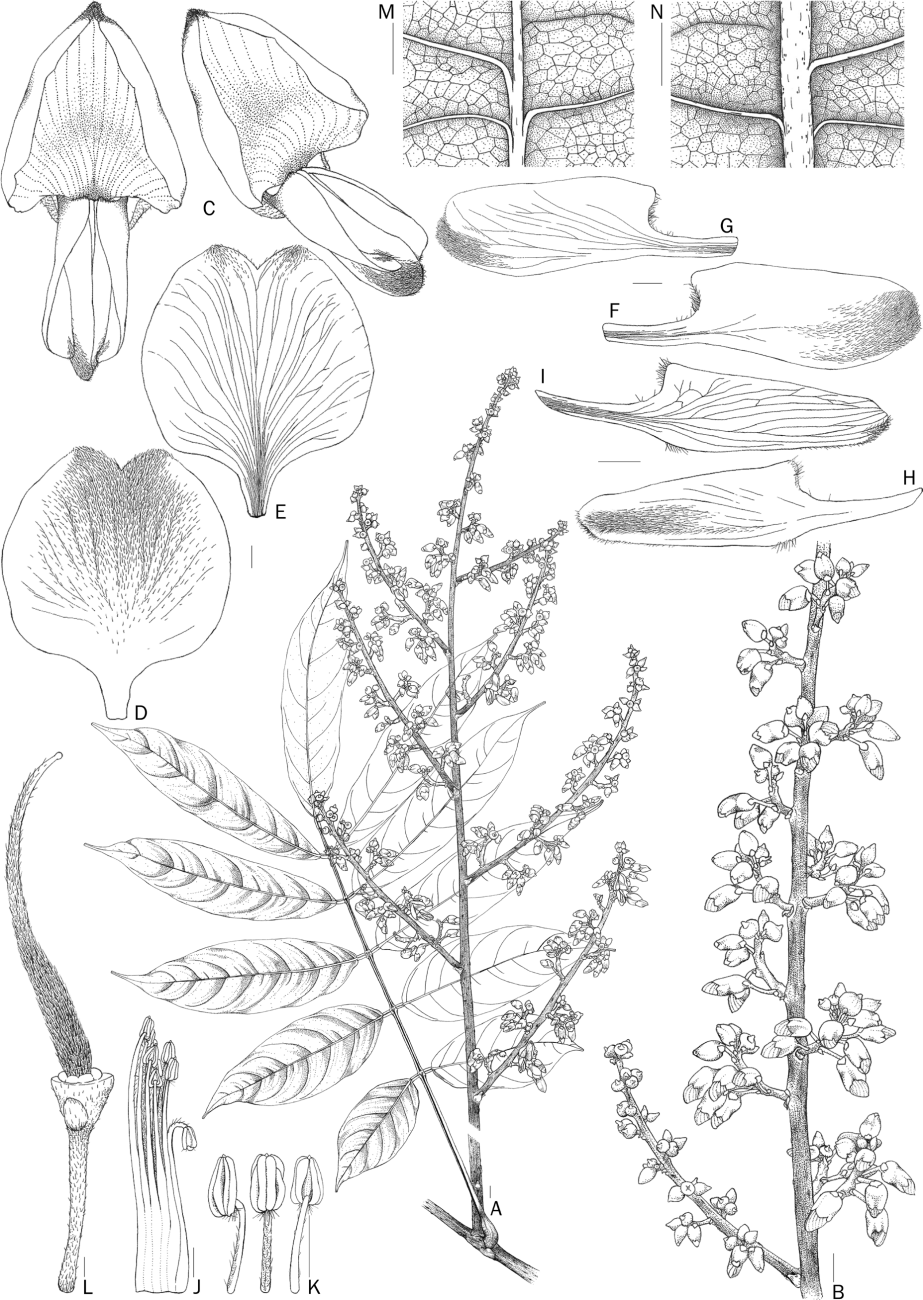
*D.rubricosta***A** inflorescence with leaf **B** close-up of inflorescence **C** flowers (top and side view) **D** outer surface and **E** inner surface of standard petal **F** outer surface and **G** inner surface of keel petals **H** outer surface and **I** inner surface of wing petals **J** staminal sheath **K** anthers **L** pistil **M** adaxial and **N** abaxial leaflet surfaces. Drawn by Punvarit Boonprajan from C. Leeratiwong 19-1666 (BKF). Scale bars: 5 mm (**A, B**); 1 mm (**C–N**).

**Figure 4.**
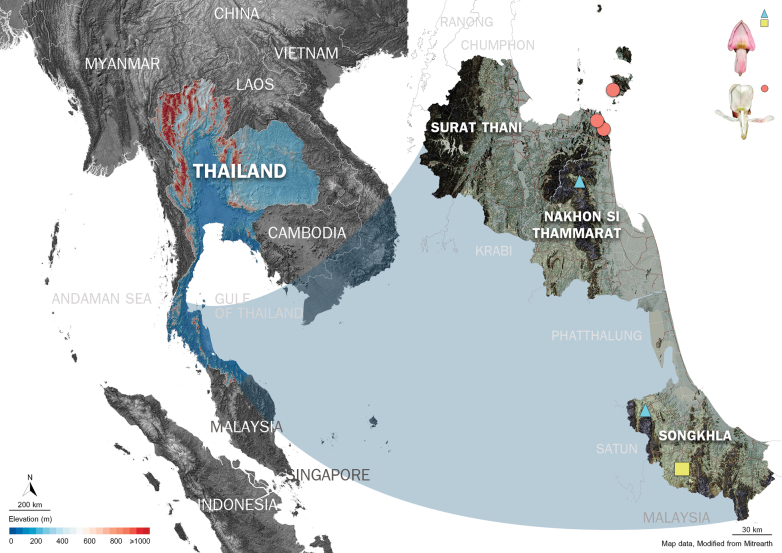
Collection sites of the new *Derris* samples, represented by a yellow square (flowering specimen) and light-blue triangle (specimen without flowers) and *D.pubipetala* (orange circles) in Peninsular Thailand.

Three putative new *Derris* samples were morphologically studied and photographed as shown in Figs 1–3. They were collected from three localities in Songkhla and Nakhon Si Thammarat provinces (Fig. 4, a yellow square and 2 light-blue triangles). Their overall vegetative morphology is the same and they presumably represent a single species. The most remarkable characteristic found in all three samples is the reddish midribs of the mature leaflets. Only the sample collected from Pha Dam Forest Ranger Unit in Songkhla province had flowers. Morphologically, the most similar species in Peninsular Thailand is *D.pubipetala*, although this differs by several morphological characters. In general, the putative new *Derris* samples have paler bark and more leaflets per leaf, and the inflorescence is longer than that of *D.pubipetala*. Shape, curvature, and the lower auricle of the wing petals are also different. Stamens of the new *Derris* are hairy on the free part of their filaments and, more importantly, hairs at the base of the anthers are not present in *D.pubipetala* or any other species of *Derris*. Comparisons between the morphological characters of the new *Derris* and *D.pubipetala* are summarised in Table 3.

**Table 3.** Comparative morphological characters of the new *Derris* and *D.pubipetala*.

Morphological characters	Species	
	*Derris* sp. nov.	*D.pubipetala*
**Vegetative parts**		
Colour of roots	brownish to black-gray	slightly pinkish or reddish
Colour of bark	pale brownish-gray to gray	dark reddish-brown
Colour of leaves when young	reddish	light green to brownish
Number of leaflets per leaf	9–11	5–9
Colour of midrib of leaves when mature	reddish	green
Adaxial leaf surface	glabrous except for slightly strigose along midrib and lateral veins	glabrous to slightly strigose
**Reproductive parts**		
Length of inflorescence (cm)	40–50	5–28
Position of bracteoles	at the base of calyx tube	on pedicel
Colour of corolla	pale pink to pink	white
Shape of wing petals	elliptic to narrowly ovate	elliptic to semi-hastate
Apex of wing petals	obtuse	rounded
Upper auricle of wing petals	indistinct, 0.5–0.9 mm long	evident, 1–2.5 mm long
Lower auricle of wing petals	absent	present
Curvature of wing petals	straight	curved backward
Apex of keel petals	retuse	rounded
Anthers with some basal hairs	present	absent
Free part of filaments with hairs	present	absent
Shape of floral disc	indistinct or more or less 10-lobed	annular
Indumentum on style	sericeous at the base and gradually becoming glabrous apically	glabrous

### ﻿Anatomical study of leaves/leaflets

**Figure 5.**
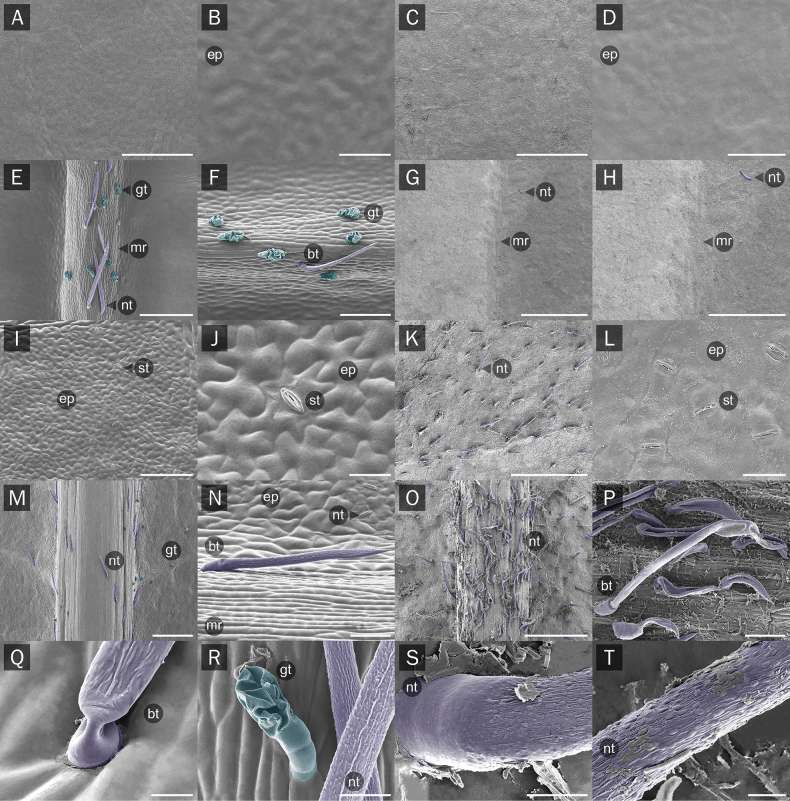
Scanning electron microscope (SEM) photographs showing leaflet micromorphology **A–D**) adaxial and **I–L** abaxial surfaces of leaflet lamina **E–H** adaxial and (**M–P**) abaxial surfaces of midrib **Q–T** non-glandular and glandular trichomes **A, B, E, F, I, J, M, N, Q, R** the new *Derris* samples and **C, D, G, H, K, L, O, P, S, T***D.pubipetala*; bt = bicellular non-glandular trichome; ep = epidermal cell; gt = glandular trichome; mr = midrib; nt = non-glandular trichome; st = stoma. Scale bars: 5 µm (**T**); 10 µm (**Q, S**); 20 µm (**J, L, R**); 50 µm (**B, D, N, P**); 100 µm (**F, I**); 200 µm (**E**); 500 µm (**A, C, H, K, M, O**); 1000 µm (**G**).

**Figure 6.**
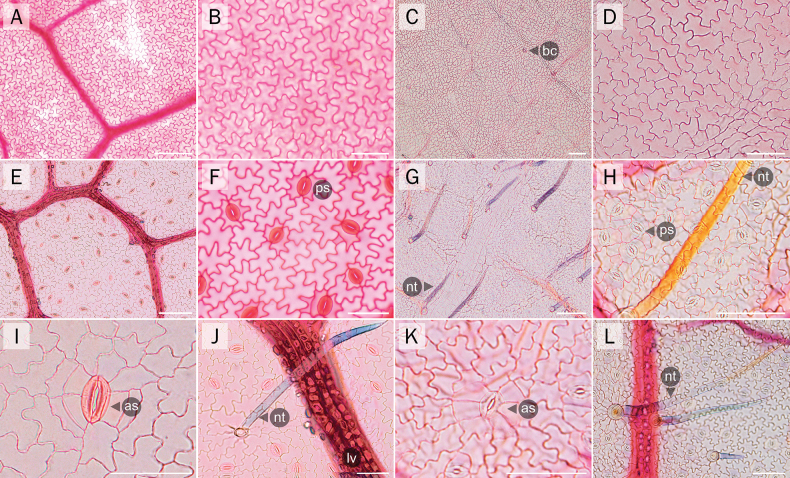
Comparative anatomical characters of the leaflet epidermis **A–D** adaxial leaflet epidermis **E–H** abaxial leaflet epidermis **A, B, E, F, I, J** the new *Derris* samples and **C, D, G, H, K, L***D.pubipetala*. as = anomocytic stomata; bc = basal cell of trichome; nt = non-glandular trichome; ps = paracytic stomata. Scale bars: 50 µm (**B, D, F, H–L**); 100 µm (**A, C, E, G**).

**Figure 7.**
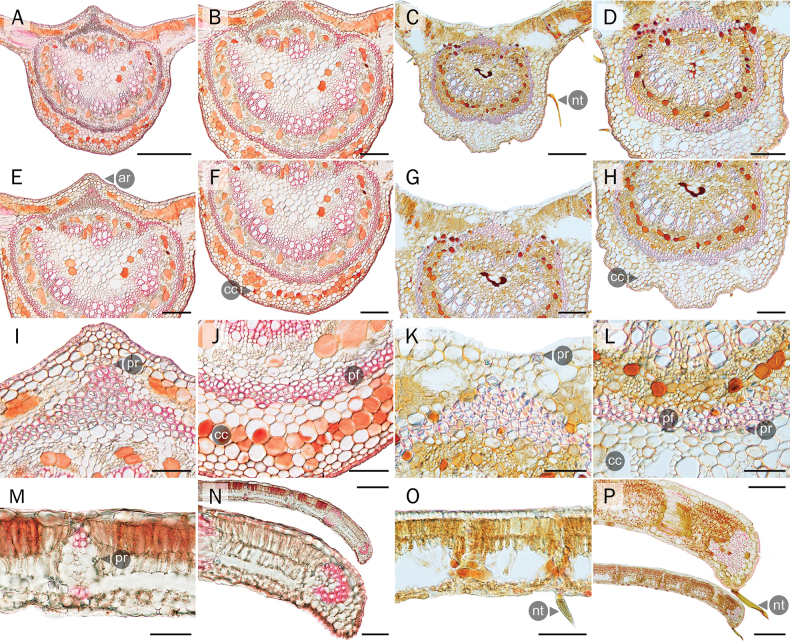
Comparative anatomical characters of leaflet transverse sections **A–L** midrib **M, O** leaflet blade and **N, P** leaflet margin **A, B, E, F, I, J, M, N** the new *Derris* and **C, D, G, H, K, L, O, P***D.pubipetala*. ar = adaxial ridge; cc = cortical cells; nt = non-glandular trichome; pf = perivascular fibers; pr = prism. Scale bars: 50 µm (**M, N** (below), **O, P** (above)); 100 µm (**I–L**); 200 µm (**B, D, E–H, N** (above), **P** (below)); 500 µm (**A, C**).

As seen in *D.pubipetala*, all samples of the putative new *Derris* (Table 1) showed no significant differences in their leaf anatomy. Micro-morphological and anatomical characteristics are summarised in Table 4 and presented in Figs 5–7.

**Table 4.** Comparison of selected anatomical characters of the new *Derris* species and *D.pubipetala*. [Quantitative data; Mean value ± standard deviation (SD)].

Anatomical characters	Species	
	*Derris* sp. nov.	*D.pubipetala*
**Leaflet epidermis**		
**Adaxial leaflet surface**		
Pattern of epidermal cell walls	undulate	undulate
Width of epidermal cell walls (µm)	41.53±5.52	35.73±3.94
Length of epidermal cell walls (µm)	55.05±6.50	43.22±4.71
Indumentum	glabrous, slightly strigose along midrib and lateral veins	thinly strigose
Ratio of epidermal cell walls (width: length)	0.77±0.20	0.83±0.09
**Abaxial leaflet surface**		
Pattern of epidermal cell walls	undulate	undulate
Width of epidermal cell walls (µm)	37.28±2.71	21.91±2.42
Length of epidermal cell walls (µm)	56.60±6.81	31.15±10.43
Ratio of epidermal cell walls (width: length)	0.66±0.12	0.78±0.36
Types of stomata	Ps, rarely As	Ps, rarely As
Width of stomata (µm)	17.47±1.01	12.43±0.73
Length of stomata (µm)	22.95±1.03	15.04±0.52
Ratio of stomatal (width: length)	0.76±0.04	0.82±0.07
Stomatal density (per mm^2^)	104.66±3.05	230±15.62
Number of epidermal cells per unit area (mm^2^)	511.33±38.27	1161.33±20.42
Stomatal Index (SI)	17.02±0.75	16.52±1.04
Width of guard cell (µm)	8.24±0.76	5.17±0.08
Length of guard cell (µm)	22.65±1.46	15.41±0.34
Ratio of guard cell (width: length)	0.36±0.01	0.33±0.00
Indumentum	glabrous to thinly strigose	strigose
Types of indumentum	Ut	Bt
Length of indumentum hairs (µm)	204.70±80.00	ST = 60.10±16.26 MT = 265.02±39.15 LT = 457.25±30.70
Ratio of epidermal cell width on adaxial leaflet surface to epidermal cell width on abaxial leaflet surface (width: width)	1.11±0.07	1.64±0.25
Ratio of epidermal cell length on adaxial leaflet surface to epidermal cell length on abaxial leaflet surface (length: length)	0.97±0.07	1.54±0.72
**Leaflet transverse sections**		
**Transverse section of Midribs**		
Outline of midribs	convex on the abaxial surface with an adaxial ridge	convex on the abaxial surface, either flat or slightly concave on the adaxial surface
Width of midrib (µm)	1200.15±234.88	616.44±85.36
Height of midrib (µm)	1241.50±244.61	593.48±80.23
Ratio of midrib (width: height)	0.96±0.00	1.04±0.10
Height of epidermal cells on adaxial leaflet surface (µm)	9.96±3.59	13.9±0.92
Height of epidermal cells on abaxial leaflet surface (µm)	8.55±0.38	8.41±0.72
Width of vascular bundle (µm)	1067.22±240.35	481.48±64.20
Height of vascular bundle (µm)	923.20±240.47	406.98±52.31
Ratio of vascular bundle (width: height)	1.16±0.05	1.18±0.08
Types of indumentum	UT, BT, GT	BT
**Transverse section of Leaflet Blades**		
Thickness of leaflet blade (µm)	204.27±37.90	150.35±8.43
Height of epidermal cells on adaxial leaflet surface (µm)	23.49±5.80	11.59±1.03
Height of epidermal cells on abaxial leaflet surface (µm)	13.06±0.99	9.22±1.49
Thickness of palisade mesophyll (µm)	105.14±19.70	59.62±7.51
Thickness of spongy mesophyll (µm)	64.48±20.24	69.85±5.19
Thickness ratio of palisade mesophyll to spongy mesophyll	1.68±0.31	0.85±0.13
Number of palisade mesophyll layers	2 or rarely 3	2
**Transverse section of Leaflet Margin**		
Outline of leaflet margin	downward	downward
Thickness of leaflet margin (µm)	143.98±5.86	138.04±4.19
Height of epidermal cells on adaxial leaflet margin (µm)	10.84±1.33	14.10±2.61
Height of epidermal cells on abaxial leaflet margin (µm)	7.73±1.19	10.34±0.21
Thickness of palisade mesophyll (µm)	74.57±6.01	49.54±7.35
Thickness of spongy mesophyll (µm)	31.88±2.27	54.42±11.82
Thickness ratio of palisade mesophyll to spongy mesophyll	2.35±0.34	0.95±0.31
Number of palisade mesophyll layers	2	2
Angle of leaflet margin curvation	9.16±1.68	16.01±3.04

Note: As = anomocytic stomata; BT = bicellular non-glandular trichome; GT = glandular trichome; LT = long trichome; MT = medium trichome; Ps = paracytic stomata; ST = short trichome; UT = unicellular non-glandular trichome.

**Leaf epidermis** (Fig. 6A–L); Leaf epidermal cells of the three new *Derris* samples and those of *D.pubipetala* were quite similar on both leaflet surfaces, i.e., lobed or jigsaw-like in shape because of their undulate anticlinal cell walls (slightly more deeply undulate in the new *Derris* samples). The width and length of the epidermal cells on both surfaces ranged between 21.91–41.53 µm and 31.15–56.60 µm, respectively. Ratios of epidermal cell size (width: length) of the new *Derris* species were 0.77 (±0.20) for the adaxial and 0.66 (±0.12) for the abaxial surface, whereas the ratios of *D.pubipetala* were 0.83 (±0.09) for adaxial and 0.78 (±0.36) for abaxial surfaces, respectively. Leaflets of all taxa were hypostomatic with commonly paracytic and rarely anomocytic stomata. The width and length of stomata in the new species of *Derris* (w = 17.47±1.01 µm; l = 22.95±1.03 µm) was greater than in *D.pubipetala* (w = 12.43±0.73 µm; l = 15.04±0.52 µm), whereas the stomatal density of *D.pubipetala* (230±15.62 per mm^2^) was higher than for the new *Derris* (104.66±3.05 per mm^2^). The stomatal index of the new species was also higher (17.02±0.75) than that of *D.pubipetala* (16.52±1.04). Guard cell size of the new *Derris* was c. 22.65 µm long and c. 8.24 µm wide (ratio = 0.36±0.01). For *D.pubipetala*, guard cells were clearly smaller, c. 15.41 µm long and c. 5.17 µm wide (ratio = 0.33±00). Only unicellular non-glandular trichomes occurred on both leaflet surfaces in the new *Derris*. In *D.pubipetala*, unicellular non-glandular trichomes occurred only on the adaxial side and three different lengths (large: more than 400 µm; medium: > 100–400 µm, and small: less than 100 µm) of bicellular non-glandular trichomes were found only on the abaxial surface. No significant differences in leaflet epidermis were found among the three accessions of the new *Derris* or among populations of *D.pubipetala*.

**Transverse sections** (Fig. 7A–P); the adaxial leaflet surface midrib of the new *Derris* samples is convex, while it is flat or slightly concave in *D.pubipetala*. The midrib transverse section of the new *Derris* species is larger (w = 1200.15±234.88 µm; h = 1241.50±244.61 µm) than in *D.pubipetala* (w = 616.44±85.36 µm; h = 593.48±80.23 µm). The height of the epidermal cells on the adaxial and abaxial surface of the midrib ranged from 8.41–13.9 µm. in both species. The midrib of both taxa shows an elliptic or semi-circular shaped vascular bundle, surrounded by perivascular fibers. Inside the vascular bundle, vascular tissue is presented in two groups; smaller and fan-shaped on the adaxial surface and larger and horseshoe-shaped on the abaxial surface. The vascular bundle of all samples of the new *Derris* was larger (w = 1067.22±240.35 µm; h = 923.20±240.47 µm) than was found in *D.pubipetala* (w = 481.48±64.20 µm; h = 406.98±52.31 µm). Bicellular non-glandular and glandular trichomes were restricted to the midrib of the new *Derris* (Fig. 5E, F, M, N). In *D.pubipetala*, on the other hand, only bicellular non-glandular trichomes were found (Fig. 5O, P, 7C, D).

All taxa had dorsiventral leaves (Fig. 7M, O), with palisade mesophyll adaxially and spongy mesophyll abaxially. The new *Derris* samples have a greater leaflet thickness (204.27±37.90 µm) than in *D.pubipetala* (150.35±8.43 µm). The epidermal cell heights on the adaxial and abaxial surfaces of leaflet blades in all studied samples were between 9.22–23.49 µm. Furthermore, the mesophyll of the new *Derris* has a thicker 2–3-layered palisade parenchyma (105.14±19.70 µm) than in *D.pubipetala* (59.62±7.51 µm). However, spongy parenchyma in *D.pubipetala* was thicker (69.85±5.19 µm) than in samples of the new *Derris* (64.48±20.24 µm). Spongy mesophyll of *D.pubipetala* consists of variously-shaped, loosely arranged cells with larger intercellular air spaces than in the new *Derris*, which possessed fewer and smaller intercellular air spaces.

Transverse sections of the leaflet margin in all samples revealed them as being slightly revolute (Fig. 7N, P). The leaflet margin of the new *Derris* was thicker (143.98±5.86 µm) than in *D.pubipetala* (138.04±4.19 µm). Height of the epidermal layer on the adaxial and abaxial surfaces ranged from 7.73–14.10 µm. Thickness of palisade and spongy parenchyma at the leaflet margin of the new *Derris* samples and *D.pubipetala* were (74.57±6.01 vs. 31.88±2.27 µm) and (49.54±7.35 vs. 54.42±11.82 µm), respectively.

Leaflet transverse sections of all samples demonstrated the accumulation of prismatic crystals, generally associated with vascular bundles, and particularly in the leaflet midribs. Rhomboidal prisms and styroid prisms were two types of crystal observed in the leaflets of the new *Derris* species, whereas only rhomboidal di-pyramid prisms were seen in *D.pubipetala*. The reddish substance present in cortical parenchyma cells of the leaflet midrib on the abaxial leaflet surface is a unique characteristic observed only in the new *Derris*.

### ﻿Chemical fingerprinting

**Figure 8.**
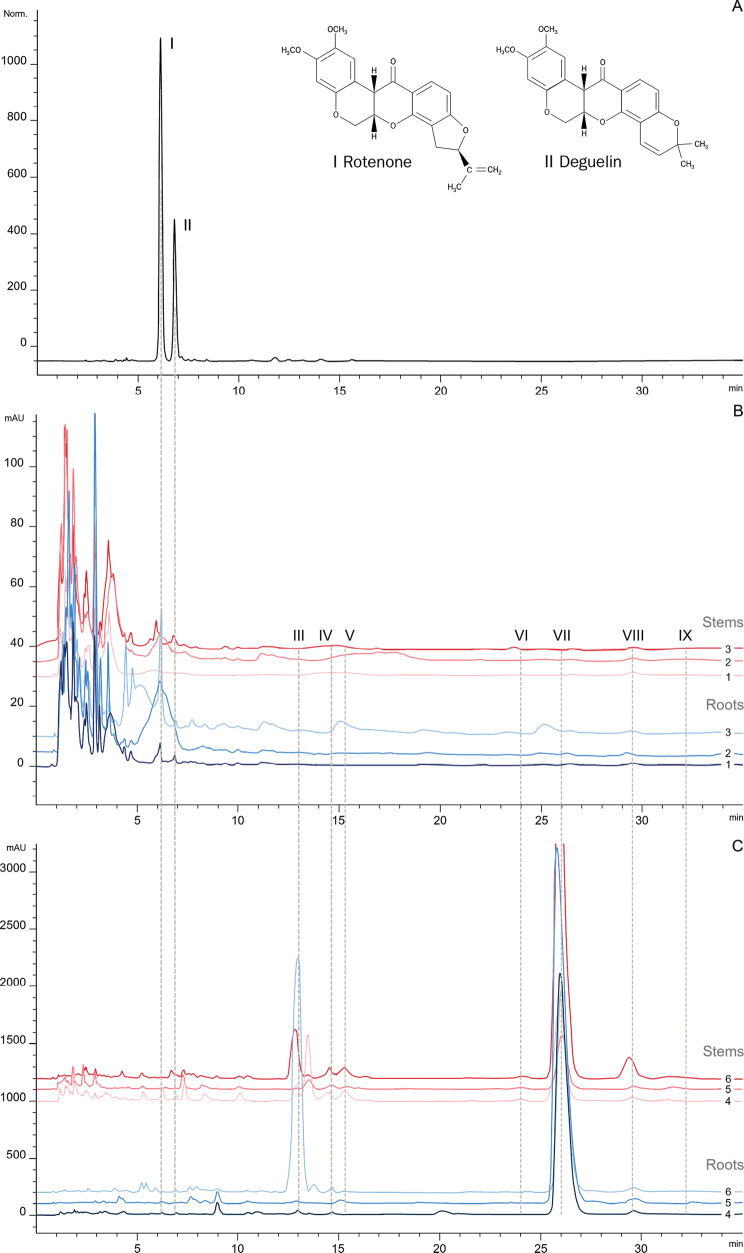
High-performance liquid chromatography (HPLC) chromatograms of the new *Derris* samples and *D.pubipetala* roots (the lower, blueish lines) and stems (the upper, reddish lines) extracts **A** two standard compounds **B** The new *Derris* species samples **C***D.pubipetala*; Each Arabic numeral represents the accession number of each sample presented in Table 1. Each Roman numeral represents each peak of HPLC chromatograms (chemical compound) with its retention time, i.e., (**I**) RT, 6.1 min; (**II**) DG, 6.7 min; (**III**) UK1, 13.3 min; (**IV**) UK2, 14.4 min; (**V**) UK3, 15.3 min; (**VI**) UK4, 24.0 min; (**VII**) UK5, 26.1 min; (**VIII**) UK6, 29.5 min; (**IX**) UK7, 32.2 min; RT = rotenone; DG = deguelin; UK = unknown.

HPLC chromatograms (Fig. 8) revealed intra- and inter-specific differences in chemical compounds of the root and stem crude extracts. Under optimal HPLC conditions, the peaks of two chemical markers showed acceptable resolution (Fig. 8A). The standard peaks of rotenone and deguelin were detected at 6.1 (peak I) and 6.7 (peak II) min, respectively. Most of the HPLC chromatograms (8 of the 12) of the new *Derris* (Fig. 8B) and *D.pubipetala* (Fig. 8C) displayed two common chemical markers, i.e., Rotenone (I) and Deguelin (II). Exceptions were noted in the *Derris* new species’ accession no. 2 (both root and stem extract), in the stem extract of the new *Derris* accession no. 1, and in the stem extract of *D.pubipetala* accession no. 5. Peaks of unknown chemical compounds (III–XI) were detected only in *D.pubipetala*, and were absent in all samples of the new *Derris*.

### ﻿Molecular phylogeny

**Figure 9.**
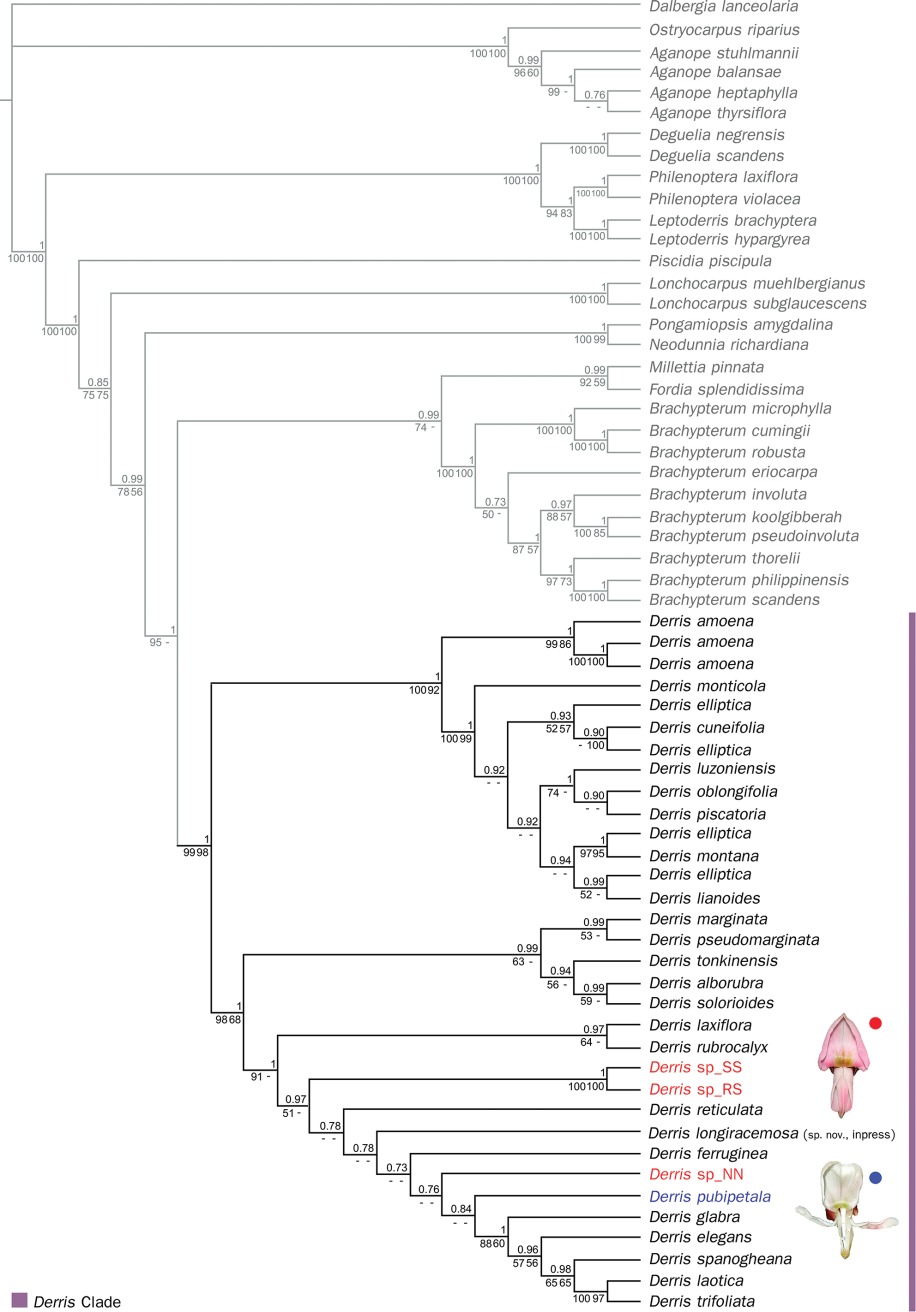
Consensus tree from Bayesian Inference analysis (BI) of concatenated two plastid and one nuclear dataset. The posterior probabilities (PP) are shown above the branches. Bootstrap percentage support values of Maximum likelihood (MLBS) and Maximum parsimony (MPBS) are shown below the branches, respectively (- = MPBS and/or MLBS < 50%). Abbreviations and numbers after the scientific names indicate locality codes following Table 1: NN = Nopphitam district, Nakhon Si Thammarat province; RS = Rattaphum district, Songkhla province; SS = Sadao district, Songkhla province, Thailand. Red and blue lettering highlight the position of the three samples of the new *Derris* and *D.pubipetala*, respectively.

A phylogeny based on combined nuclear and chloroplast sequences using three analyses (MP, ML, and BI) demonstrated different degrees of resolution and support, but with compatible tree topologies. According to the cladogram of the Bayesian analysis (Fig. 9), the clade of the genus *Derris* has very high support (PP 1.00, MLBS 99%, MPBS 98%) and is sister to the clade consisting of members of the genus *Brachypterum* including *Millettiapinnata* (L.) Panigrahi and *Fordiasplendidissima* (Blume ex Miq.) Buijsen. The *Derris* clade is divided into several subclades with varying clade support.

Samples of the putative new *Derris* species from three separate populations, however, did not form a clade in the Maximum Likelihood and Bayesian analyses because the sample from Nakhon Si Thammarat province (NN in Fig. 9) was separate from the other two samples from Songkhla province (SS and RS). Those two samples formed a strongly supported clade in all three analyses (PP 1.00, MLBS 100%, MPBS 100%). The clade emerged as sister to the clade consisting of *D.elegans* Benth., *D.ferruginea* Benth., *D.glabra* Sirich., *D.laxiflora* Benth., *D.laotica* Gagnep., *D.laxiflora* Benth., *D.longiracemosa* (sp. nov., in press), *D.reticulata* Craib, *D.rubrocalyx* Verdc., *D.trifoliata* and the new taxon sample from Nakhon Si Thammarat province, with high PP (0.97) but low MLBS (51%) and MPBS (< 50%) clade support. A slightly different topology emerged in the Maximum Parsimony (MP) analysis, where the three new species samples formed a moderately supported clade (MPBS 72%, see Suppl. material 2).

## ﻿Discussion

Based on morphological, anatomical, phytochemical, and phylogenetic investigations of the new *Derris* samples, it is evident that they represent a novel and distinct species.

Macro- and micro-morphologically, the new *Derris* is similar to *D.pubipetala*. The two species also share the same distribution area and thrive in very similar habitats. (Fig. 4; Southern Thailand) (Sirichamorn et al. 2014a; Sirichamorn 2020). The reddish colour of the leaflet midrib is the most distinctive vegetative character of the new species.

Microscopic studies of leaflets revealed additional unique characters of the new species. Small glandular trichomes are distributed mainly on the midrib on both surfaces of the leaflets; together with uni- and bicellular non-glandular hairs (Fig. 5E, F, M, N, Q, R). Glandular trichomes usually function as herbivore deterrents and protect against oxidative stress due to their ability to produce, store, or secrete chemical substances, as reported for many plants (Peiffer et al. 2009; LoPresti 2016; Murungi et al. 2016; Stojičić et al. 2016; Zhao and Chen 2016; Tozin and Rodrigues 2017; Jachuła et al. 2018; Li et al. 2018), but their presence in species of *Derris* is quite rare. A few species, e.g., *D.elegans*, *D.elliptica* (Wall.) Benth., *D.ferruginea*, and *D.pubipetala*, have been reported as having hairy leaves, but only non-glandular trichomes.

Several characteristics of the reproductive parts of the new species differ from *D.pubipetala*. For example, prominent hairs on stamen filaments, below the anthers, have never been reported for any other *Derris* species, including *D.pubipetala*. Hairs at the base of the anthers do occur, however, in some members of the genus *Millettia* Wight & Arn., e.g., *M.extensa* (Benth.) Benth. ex Baker and *M.pinnata* (Mattapha 2020; Sirichamorn 2020). The function of these stamen hairs needs further investigation.

Pod traits, such as the number of wings on the margin of the pods or the density of the hairs covering the pod surface, are among the most important characters for identifying members of *Derris*. Most species of *Derris* have two-winged pods. For example, pods of *D.pubipetala* are two-winged and densely covered with golden-brown hairs (Sirichamorn 2020). Based on the phylogenetic position of the new species, we may assume that its pods are also two-winged, although the collection of mature fruits is needed to confirm this.

Epidermal cell size of the new *Derris* is larger than in *D.pubipetala*, notably on the lower leaflet surface. Additionally, larger stomata were also observed. The Stomatal Index (SI) of *D.pubipetala* is lower because the size of epidermal cells and stomata are both smaller than in the new *Derris*.

Leaflet thickness of the new *Derris* samples is positively correlated with the height of the epidermal cells and palisade mesophyll, which were generally greater than in *D.pubipetala*. In contrast, the height of the spongy mesophyll in *D.pubipetala* is greater. Palisade mesophyll on the adaxial surface of the leaflets is the primary site of photosynthesis and cells of this layer are directly exposed to light. The plants growing in more sunlight may develop a thicker palisade layer, not only to increase photosynthesis but also to prevent the deeper leaflet tissue from sunlight damage (Terashima et al. 2011; Gotoh et al. 2018).

Our study presents the first report of HPLC fingerprinting of the new *Derris* species and *D.pubipetala*. Results showed peaks of unique chemical markers that can be used for the rapid identification of *Derrisrubricosta* and *D.pubipetala*. Intra-specific variation of chemical constituents was noted among populations, a phenomenon commonly found in other plant studies (Chen et al. 2015; Saraf and Shinede 2016, 2018). Production and accumulation of phytochemicals are not only dependent on plant species, but also on environmental factors (Qaderi et al. 2023).

Cladograms from all phylogenetic analyses, based on 62 accessions, exhibit similar topologies to the previous study by Sirichamorm et al. (2012a) (Fig. 9). *Derris* is strongly supported as monophyletic. The phylogenetic positions of three newly collected accessions demonstrated that they are members of the genus *Derris*. However (and surprisingly), the samples apparently are not representative of a single taxon, at least based on molecular analysis. The three accessions, of what we are recognizing as *D.rubricosta*, from three localities separated into two groups, i.e., one from Songkhla province (consisting of *Derris* sp_SS and *Derris* sp_RS) and the other from Nakhon Si Thammarat province (*Derris* sp_NN). Nevertheless, vegetative morphology (and leaflet anatomy) of these three accessions was almost identical. It is possible that two taxa should be recognized but this will only be resolved when more reproductive parts are collected.

Here we preliminarily accept the two accessions from Songkhla province (one with flowers, *Derris* sp_SS, and the other without reproductive parts, *Derris* sp_RS) as a species new to science. The accession from Nakhon Si Thammarat province (*Derris* sp_NN) is considered as a taxon of unknown status to be further reviewed pending development of flowers.

### ﻿Taxonomic treatment

#### ﻿ Derris rubricosta

Taxon classificationPlantaeFabalesFabaceae

Boonprajan & Sirich. sp. nov.

2B1F44BC-155E-5FA4-8C84-C4492A053A31

urn:lsid:ipni.org:names:77334641-1

Figs 1 , 2 , 3

##### Type.

Thailand. Songkhla, Sadao district, Padang Besar sub-district, Pha Dam Forest Ranger Unit, Ton Nga Chang Wildlife Sanctuary, c. 150 m elevation, GPS coordinate 6°47'16.7"N, 100°13'51.8"E, 22 January 2019, *C. Leeratiwong 19-1666* (holotype BKF!; isotypes K!).

##### Diagnosis.

The species has several autapomorphies distinguishing it from other *Derris* species. It is the only species that has reddish midribs on the lower surface of mature leaflets. Its style is sericeous at the base and gradually becomes glabrous apically (vs. thinly hairy at the base and mostly glabrous throughout in other *Derris* species). *Derrisrubricosta* has prominent hairs below the anthers (vs. glabrous anthers in all other *Derris* species). It is morphologically similar to *D.pubipetala* Miq., but differs by its leaflet midrib colour (reddish vs. green), number of leaflets er leaf (9–11 vs. 5–9), colour of its corolla (pale pink to pink vs. white), wing petal margin (straight vs. revolute), stamen filament indumentum (sparsely hairy vs. glabrous), indumentum presence below the anthers (present vs. absent), floral disc shape (indistinct to more-or-less 10-lobed vs. annular).

##### Description.

Woody climber. ***Bark*** pale brownish-gray to gray, lenticellate. ***Leaves*** with 9–11 leaflets, reddish when young, chartaceous to sub-coriaceous. ***Stipules*** caducous (not present on herbarium specimens); petiole 6–10.8 cm long, grooved above, thinly strigose to almost glabrous; rachis 10–18 cm long, grooved above, thinly strigose to glabrous; pulvinus 9–15 mm long, thinly strigose; stipellae absent; terminal leaflet elliptic to obovate or narrowly oblong, 10–18.3 × 3.0–4.3 cm, length/width ratio 3.2–4.3, base cuneate, apex acuminate, acumen 7.2–18 mm long, emarginate, upper surface glabrous but slightly strigose along midrib and lateral veins, lower surface glabrous to sparsely strigose along midrib, lateral veins and lamina, sometimes slightly strigose to glabrous at the margin, midrib (reddish on fresh mature leaflets) and secondary veins slightly raised or flat above, raised below, 7–9 each side of the midvein, 0.7–2.7 cm a part, curving towards the apex and almost reaching the margins, sometimes anastomosing near the margins, tertiary venation reticulate, pulvinus 5.0–6.5 mm long, sparsely strigose; lateral leaflets mostly like the terminal one, narrowly elliptic to obovate, rarely ovate, 10–18.8 × 3–4.4 cm, length/width ratio 3–4.3; pulvinus of petiolules 4.5–6.5 mm long, sparsely strigose to sericeous. ***Inflorescence*** a pseudoracemes or pseudopanicle, axillary or terminal, 40–50 cm long; peduncle 2–7 cm long, lenticellate, strigose; bracts subtending inflorescence triangular, 2–2.6 × 1.8–2.5 mm, outside with some hairs at base and along margin apically, inside glabrous; bracts subtending lateral branches triangular, ovate, 2–2.5 × 1.8–2.4 mm, outside with some hairs at base and along margin apically, inside glabrous; lateral branches 3.4.–15 cm long, sparsely strigose at base, lenticellate; bracts subtending brachyblasts ovate-triangular, 1–2.5 × 0.8–1.4 mm, outside with some hairs at base and along margin apically, inside glabrous. ***Brachyblasts*** knob-like to elongate-cylindrical, 1–12 mm long, 2-flowered, strigose; bracts subtending flowers ovate-triangular, 0.7–0.9 × 0.7–1 mm, outside sparsely hairy at base and along margin, inside glabrous; pedicels 3.5–5.2 mm long, strigose; bracteoles 2, at base of calyx, ovate, semi-circular, orbicular to narrowly triangular, 0.6–0.9 × 0.5–0.7 mm, outside sparsely strigose, with some hairs along the margin, inside glabrous. ***Calyx*** red- to maroonish, cup-shaped 3.4–4.2 mm long, outside sparsely strigose, with some hairs along the margin, inside glabrous; tube 3–3.2 mm long; upper lip with 2 short lobes, 0.2–0.4 × 1.5–2 mm; lateral lobes short-triangular, 0.2–0.6 × 0.7–1 mm; lower lobes triangular 0.3–1.3 × 0.8–1.2 mm. ***Corolla*** pale pink to pink; standard orbicular or broadly ovate, with a greenish-yellow spot at the base on the inner surface, 8.5–10 × 8.6–9.3 mm, apex emarginate, basal callosities absent, outside hairy from the middle part to apex, inside with some hairs near apex, claw 1.5–2.8 mm long; wings elliptic to narrowly ovate, 7.3–8.2 × 3.1–4 mm, apex obtuse, upper auricle indistinct, pubescent, 0.5–0.9 mm long, lower auricle absent, lateral pocket 1.4–2.2 mm long, outside hairy in the middle part of the petal to the apex, inside hairy near the apex, claw 1.8–3.5 mm long; keels boat-shaped 7–7.8 × 2.3–3 mm, apex retuse, upper auricle pubescent, 0.5–1 mm long, lower auricle absent, lateral pocket 1–2.1 mm long, outside and inside hairy near the apex, sometimes also with sparse hairs along the veins ventrally, claw 1–2.9 mm long. ***Stamens*** 10, monadelphous, 2.8–4.6 mm long, free part 1.5–3.1 mm long, sparsely hairy, anthers 0.5–0.6 × 0.2–0.3 mm, with some basal hairs. ***Disc*** indistinct, or more or less 10-lobed, glabrous. ***Ovary*** 3.5–5 mm long, sericeous; stipe usually indistinct, sericeous; style 5.6–7.4 mm long, sericeous at the base and gradually become glabrous apically. ***Pod*** and seeds unknown.

##### Phenology.

Flowering from November-February and fruiting possibly from March-April.

##### Vernacular names.

“Khruea lai leeratiwong” (เครือไหลลีรติวงศ์) means “Leeratiwong’s *Derris*”, in Thai, in honor of Associate Professor Dr. Charan Leeratiwong, who discovered and collected the type specimens.

##### Etymology.

The specific epithet refers to the striking reddish colour of the midrib of the mature leaflets which has never been found in other species of *Derris*.

##### Distribution.

Peninsular Thailand: Songkhla (Rattaphum district, Sadao district) (Fig. 4). Estimated population of more than 2,500 mature individuals were found during field survey between 2019 and 2022 in its type locality and nearby areas.

##### Habitat and ecology.

Usually near streams, in semi-shaded to fully exposed areas of tropical evergreen rainforest. The species, especially in the type locality, thrives on sandy or sandy-loam soils.

##### Proposed IUCN conservation assessment.

This new species is only known from two locations in Songkhla province. The estimated number of mature individuals might be more than 2,500 but less than 10,000. The Area of Occupancy (AOO) is about 2,000 km^2^. Although its type locality and overall distribution are located within conservation areas, the species is still threatened by human disturbance. Therefore, we provisionally assess its conservation status to be “Vulnerable (VU), B2 b(ii) c(ii)”, following the criteria of the IUCN Standards and Petitions Committee (2022, v. 15.1)

##### Representative specimens examined (paratypes).

Thailand. Songkhla: Rattaphum district, Kamphaeng Phet sub-district, 27 October 2021, *Sirichamorn Y. and Boonprajan P. YSM2021*-*15* (BKF!); Sadao district, Padang Besar sub-district, Pha Dam Forest Ranger Unit, 27 October 2021, *Sirichamorn Y. and Boonprajan P. YSM2021*-*14* (BKF!).

### ﻿Addition of *Derrisrubricosta* to the key to Thai species of *Derris*

The new taxon is inserted as couplet 7 in a modified key to species of *Derris* in the Flora of Thailand (Sirichamorn 2020; 391–392).

6 Brachyblasts variable in shape and length, usually with more than 3 flowers. Standard less than 10 mm long, rarely with basal callosities

7 Mature leaflets with reddish midribs. Stamen filament sparsely hairy. Anther base with a tuft of hairs **18. *D.rubricosta***

7’ Mature leaflets without reddish midribs. Stamen filament glabrous. Anther base glabrous

8 Pods single winged or wingless

9 Leaflets hirsute to velvety underneath; stipels present **4. *D.elegans***

9’ Leaflets glabrous underneath; stipels usually absent

10 Leaflets 3.3–7.5 × 0.9–3.5 cm; petiolules 3–5 mm long **8. *D.laotica***

10’ Leaflets 3.5–16 × 1.5–8.5 cm; petiolules 5–10 mm long **17. *D.trifoliata***

8’ Pods two-winged

11 Pods velvety or sericeous

12 Leaflets slightly strigose to velvety below, apex rounded, obtuse, or cuspidate to short-acuminate. Pods with upper wing 4–10 mm wide, lower wing 4–7 mm wide. North-eastern Thailand **6. *D.ferruginea***

12’ Leaflets usually strigose to almost glabrous below, apex distinctly acuminate. Pods with upper wing 5–9 mm wide, lower wing 2–4 mm wide. Southern Thailand **13. *D.pubipetala***

11’ Pods mostly glabrous

13 Leaflets 9–11 per leaf, narrowly obovate, base narrowly cuneate to attenuate **11. *D.monticola***

13’ Leaflets 3–7(–9) per leaf, elliptic, ovate, or obovate, base cuneate to obtuse

14 Leaflets sometimes glaucous below. Lateral veins reaching leaflet margin **2. *D.amoena***

14’ Leaflets never glaucous below. Lateral veins not reaching leaflet margin but curving toward leaf apex, sometimes forming an intramarginal vein

15 Leaflets 3–5 per leaf. Terminal leaflet distinctly longer and wider than lateral ones. Calyx glabrous outside **7. *D.glabra***

15’ Leaflets 5–7 per leaf. Terminal leaflet slightly longer but not wider than lateral the others. Calyx thinly sericeous outside **12. *D.pseudomarginata***

6’ Brachyblasts slender with 2 or 3 flowers at apex. Standard usually more than 10 mm long, usually with basal callosities

## Supplementary Material

XML Treatment for Derris rubricosta

## ﻿Additional information

### Author ORCIDs

Punvarit Boonprajan https://orcid.org/0009-0006-7251-1928

Charan Leeratiwong https://orcid.org/0000-0002-0752-4275

Yotsawate Sirichamorn https://orcid.org/0000-0002-3026-3894

### Data availability

All of the data that support the findings of this study are available in the main text or Supplementary Information.

## Supplementary materials

Supplementary material 1 Species, voucher specimen, and GenBank accession for sequence data reported in the study Data type pdf https://binary.pensoft.net/file/967535This dataset is made available under the Open Database License (http://opendatacommons.org/licenses/odbl/1.0/). The Open Database License (ODbL) is a license agreement intended to allow users to freely share, modify, and use this Dataset while maintaining this same freedom for others, provided that the original source and author(s) are credited.Punvarit Boonprajan, Charan Leeratiwong, Yotsawate Sirichamorn

Supplementary material 2 Maximum Parsimony (MP) analysis Data type jpg https://binary.pensoft.net/file/967536This dataset is made available under the Open Database License (http://opendatacommons.org/licenses/odbl/1.0/). The Open Database License (ODbL) is a license agreement intended to allow users to freely share, modify, and use this Dataset while maintaining this same freedom for others, provided that the original source and author(s) are credited.Punvarit Boonprajan, Charan Leeratiwong, Yotsawate Sirichamorn
