# Supplementary material for: ﻿From morphology to molecules: A comprehensive study of a novel Derris species (Fabaceae) with a rare flowering habit and reddish leaflet midribs, discovered in Peninsular Thailand
Source: PhytoKeys. 2024 Jan 15;237:51–77. doi: 10.3897/phytokeys.237.112860 (PMC10806910; doi:10.3897/phytokeys.237.112860)
Supplement: Supplementary material 1 — Species, voucher specimen, and GenBank accession for sequence data reported in the study [file phytokeys-237-051_article-112860__-s001.pdf]

Table S1. Species, voucher specimen, and GenBank accession for sequence data reported in the study.

Species	Voucher	GenBank accession No.			Source and Geographic regions
		trnL-F IGS	***trnK-matK***	**ITS/5.8S**	
Aganope *balansae* (Gagnep.) P.K.Lôc	Poilane 26751 (P)	JX506489	JX506601	JX506433	Vietnam, Tonkin
Aganope heptaphylla (L.) Polhill	Santisuk 688 (L)	JX506488	JX506600	JX506432	Thailand, Ranong
Aganope stuhlmannii (Taub.) Adema	Versteegh & al. 456 (L)	JX506491	JX506603	JX506435	Ivory Coast, Korhogo
Aganope thyrsiflora (Benth.) Polhill	Sirichamorn YSM 2009-22 (L)	JX506490	JX506602	JX506434	Thailand, Songkhla
Brachypterum *cumingii* (Benth.) Sirich. & Adema	Gaerlan & al. PPI 10368 (L)	JX506505	JX506618	JX506447	Philippines, Luzon
Brachypterum eriocarpa (F.C.How) Sirich. & Adema	Wang Hong 7673 (QBG)	JX506512	JX506625	JX506454	China, Yunnan
Brachypterum involuta (Sprague) Sirich. & Adema	Murray, Coveny & Bishop s.n., sheet no. NSW 409439 (L)	JX506509	JX506622	JX506451	Australia, North coast
Brachypterum koolgibberah (F.M.Bailey) Sirich. & Adema	Brass 8205 (L)	JX506511	JX506624	JX506453	Papua New Guinea, Sturt Island
Brachypterum microphylla (Miq.) Sirich. & Adema	Sirichamorn YSM 2009-16 (L)	JX506506	JX506619	JX506448	Thailand, Chumphon
Brachypterum philippinensis (Merr.) Sirich. & Adema	Elmer 14373 (L)	–	JX506627	JX506455	Philippines, Sorsogon
Brachypterum pseudoinvoluta (Verdc.) Sirich. & Adema	Streimann & Kairo NGF 27776 (L)	JX506510	JX506623	JX506452	Papua New Guinea, Morobe
Brachypterum robusta (Roxb. ex DC.) Sirich. & Adema	Sirichamorn YSM 2009-09 (L)	JX506504	JX506617	JX506446	Thailand, Lampang
Brachypterum scandens (Roxb.) Sirich. & Adema	Sirichamorn YSM 2009-01 (L)	JX506508	JX506621	JX506450	Thailand, Chon Buri
Brachypterum submontana (Verdc.) Sirich. & Adema	Takeuchi & al. 4349 (L)	JX506513	JX506626	–	Papua New Guinea, Morobe
Brachypterum thorelii (Gagnep.) Sirich. & Adema	Sirichamorn YSM 2009-03 (L)	JX506507	JX506620	JX506449	Thailand, Phrae
Dalbergia *lanceolaria* L.f.	Sirichamorn YSM 2009-02 (L)	JX506541	JX506655	JX506484	Thailand, Phrae
Deguelia *negrensis* (Benth.) Taub.	C. & F. Sastre152 (L)	–	JX506607	JX506441	Brazil
Deguelia scandens Aubl.	Granville & al. 10075 (L)	JX506495	JX506608	JX506440	French Guiana, Haut-maroni
Derris *alborubra* Hemsl.	Sirichamorn YSM 2009-14 (L)	JX506524	JX506638	JX506466	Thailand, Nakhon Nayok
Derris amoena Benth.	Sirichamorn YSM 2009-20 (L)	JX506514	JX506628	JX506456	Thailand, Surat Thani
Derris amoena Benth.	Kerr 13700 (L)	JX506515	JX506629	JX506457	Thailand, Satun

Derris amoena Benth.	Maxwell 83-11 (L)	JX506516	JX506630	JX506458	Singapore
Derris cuneifolia Benth.	Lei 612 (L)	JX506535	JX506649	JX506478	China, Hainan
Derris elegans Graham ex Benth. var. *elegans*	K. & S. Larsen KL 32828 (L)	JX506527	JX506641	JX506469	Thailand, Narathiwat
Derris elliptica (Wall.) Benth.	Sirichamorn YSM 2012-01 (SLR)	JX506533	JX506647	JX506475	Thailand, Bangkok (cutivated)
Derris elliptica (Wall.) Benth.	Kostermans 260 (L)	JX506534	JX506648	JX506477	Thailand, Kanchanaburi
Derris elliptica (Wall.) Benth.	Kantchai 101 (L)	–	–	JX506476	Thailand, Kanchanaburi
Derris elliptica (Wall.) Benth.	Sirichamorn YSM 2009-19 (L)	JX506532	JX506646	JX506474	Thailand, Surat Thani
Derris ferruginea (Roxb.) Benth.	Sirichamorn YSM 2009-13 (L)	JX506519	JX506633	JX506461	Thailand, Udon Thani
Derris glabra Sirich.	Sirichamorn YSM 2009-23 (L)	JX506521	JX506635	JX506463	Thailand, Songkhla
Derris laotica Gagnep.	Magnen, Gourgand and Châtillon s.n. (P)	JX506531	JX506645	JX506473	Cambodia
Derris laxiflora Benth.	Hu 1081	–	AF142715	AF467046	Taiwan
Derris lianoides Elmer	Ridsdale SMHI 1863 (L)	JX506539	JX506653	JX506482	Philippines, Palawan
Derris luzoniensis (Adema) Sirich. & Adema	Ridsdale, Baquiran & al. ISU 564 (L)	JX506540	JX506654	JX506483	Philippines, Luzon
Derris marginata (Roxb.) Benth.	Pierre s.n. (L)	JX506529	JX506643	JX506471	India
Derris montana Benth.	Sirichamorn YSM 2009-21 (L)	JX506536	JX506650	JX506479	Thailand, Songkhla
Derris monticola (Kurz) Prain	Kerr 1731 (L)	JX506523	JX506637	JX506465	Thailand, Chiang Mai
Derris oblongifolia Merr.	Sulit PNH 21618 (L)	JX506538	JX506652	JX506481	Philippines, Biliran island
Derris piscatoria (Blanco) Sirich. & Adema	Sulit PNH 14411 (L)	JX506537	JX506651	JX506480	Philippines, Samar
Derris pseudomarginata Sirich.	Maxwell 76-31 (L)	JX506525	JX506639	JX506467	Thailand, Chon Buri
Derris pubipetala Miq.	Maxwell 85-370 (L)	JX506520	JX506634	JX506462	Thailand, Pattani
Derris reticulata Craib	Sirichamorn YSM 2009-18 (L)	JX506518	JX506632	JX506460	Thailand, Nakhon Ratchasima
Derris rubrocalyx Verdc.	Davis 567 (L)	JX506530	JX506644	JX506472	Indonesia, Irian Jaya
Derris solorioides Sirich. & Adema	Maxwell 50-75 (L)	JX506526	JX506640	JX506468	Thailand, Nakhon Sawan
Derris spanogheana Blume ex Miq.	De Vogel 5788 (L)	JX506522	JX506636	JX506464	Indonesia, Sulawesi
Derris tonkinensis Gagnep.	Sirichamorn YSM 2009-11 (L)	JX506517	JX506631	JX506459	Thailand, Lampang
Derris trifoliata Lour.	Sirichamorn YSM 2009-06 (L)	JX506528	JX506642	JX506470	Thailand, Samut Prakan

Derris sp. SS	C. Leeratiwong 19–1666 (BKF)	OQ208831	OQ413809	OP720909	Thailand, Songkhla (Pha Dam Forest Ranger Station)
Derris sp. RS	YSM2021–15 (BKF)	OQ208832	OQ413810	OP729703	Thailand, Songkhla (Tone Prew waterfall)
Derris sp. NN	YSM2021–16 (BKF)	OQ208833	OQ413811	OP730577	Thailand, Nakhon Si Thammarat (Krung Ching waterfall)
Fordia *splendidissima* (Blume ex Miq.)	Tangah s.n.	–	AF142718	AF467048	Malaysia, Sabah
Buijsen					
Leptoderris *brachyptera* (Benth.) Dunn	Herbarium Berolinense 403 (L)	JX506498	JX506611	JX506444	Cameroon, Limbe
Leptoderris hypargyrea (Harms) Dunn	Zenker 3645 (L)	JX506497	JX506610	JX506443	Cameroon, Bipinde
Lonchocarpus *muehlbergianus* Hassl.	Hanh 2258 (L)	JX506502	JX506615	–	Paraguay, Guairá
Lonchocarpus muehlbergianus Hassl.	Tressens & al. 1992			AF467059	Argentina: Corrientes
Lonchocarpus subglaucescens Mart. ex Benth.	Hatschbach 18025 (L)	JX506501	JX506614	–	Brazil: Paraná
Lonchocarpus subglaucescens Mart. ex, Benth.	Hatschbach 41090	–	–	AF467066	Brazil
Millettia *pinnata* (L.) Panigrahi	Sirichamorn YSM 2009-25 (L)	JX506503	JX506616	JX506445	Thailand, Surat Thani
Neodunnia *richardiana* (Baillon) Geesink	Schrire 2555 (K)	–	AF142713	AF467483	Madagascar
Ostryocarpus *riparius* Hook.f.	Maesen 7524 (WAG)	JX506487	JX506599	JX506431	Benin, Ouémé
Philenoptera *laxiflora* (Guill. & Perr.) Rob.	Hu 1117	–	–	AF467488	Senegal
Philenoptera laxiflora (Guill. & Perr.) Rob.	Hu 1126	–	AF142721	–	Senegal
Philenoptera laxiflora (Guill. & Perr.) Rob.	Lykke et al 856 (L)	JX506494	–	–	Senegal: Sine Saloum
Philenoptera violacea (Klotzsch) Schrire	Busse 530 (L)	JX506493	JX506606	JX506439	German East Africa (Tanzania)
Piscidia *piscipula* (L.) Sarg.	Lavin & Luckow 5793 (TEX)	–	AF142710	AF467490	México, Veracruz
Pongamiopsis *amygdalina* (Baill.) R.Vig.	DuPuy M575 (K)	–	AF142711	AF467494	Madagascar
