# Supplementary figures and images for: ﻿From morphology to molecules: A comprehensive study of a novel Derris species (Fabaceae) with a rare flowering habit and reddish leaflet midribs, discovered in Peninsular Thailand
Source: PhytoKeys. 2024 Jan 15;237:51–77. doi: 10.3897/phytokeys.237.112860 (PMC10806910; doi:10.3897/phytokeys.237.112860)

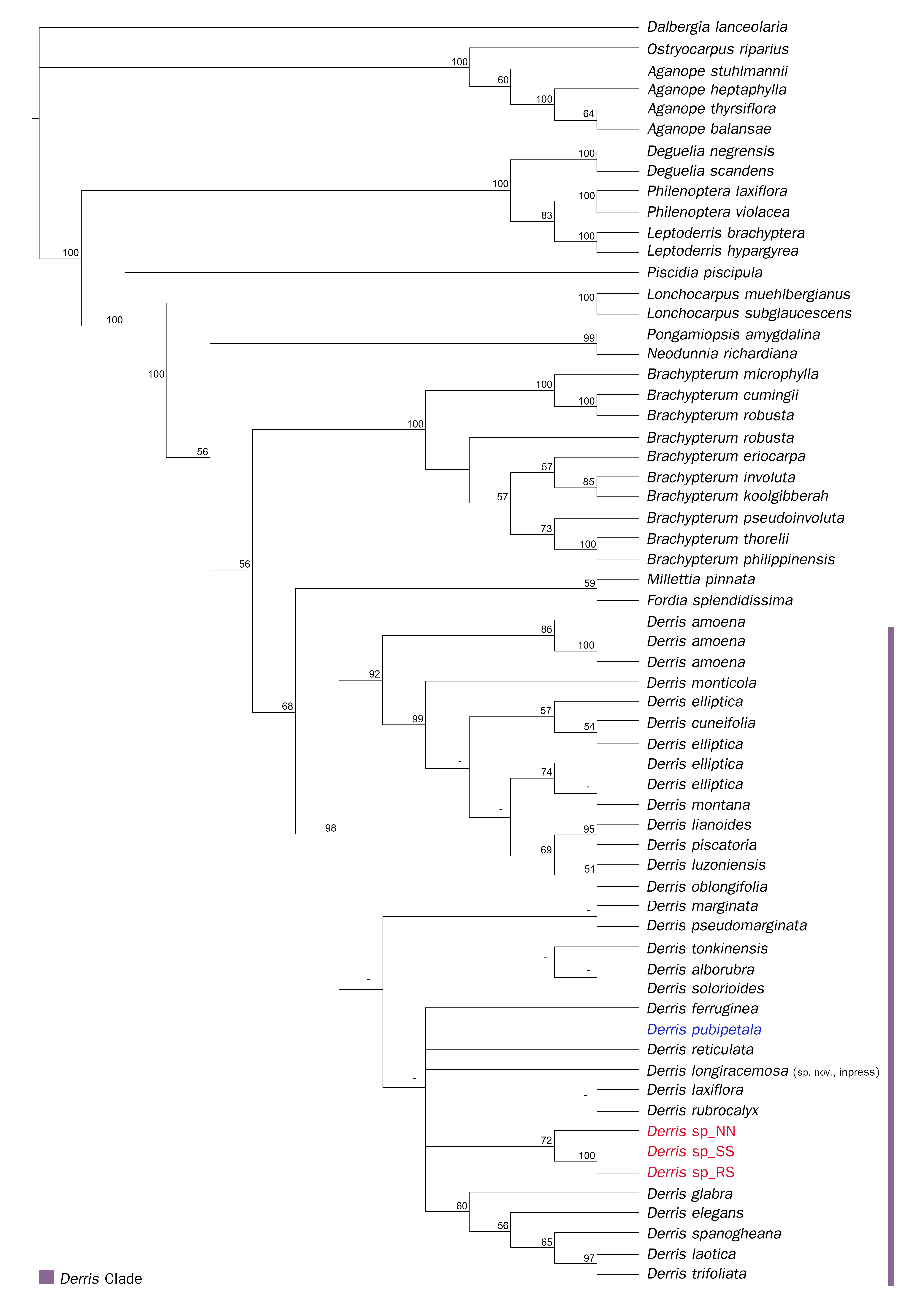

Supplement: Supplementary material 2 — Maximum Parsimony (MP) analysis [file phytokeys-237-051_article-112860__-s002.jpg]
